# Natural health products that inhibit angiogenesis: a potential source for investigational new agents to treat cancer—Part 1

**DOI:** 10.3747/co.v13i1.77

**Published:** 2006-02

**Authors:** S.M. Sagar, D. Yance, R.K. Wong

**Affiliations:** * Juravinski Cancer Centre and McMaster University (Department of Medicine), Hamilton, Ontario; † Center for Natural Healing, Ashland, Oregon, U.S.A

**Keywords:** Angiogenesis, anti-angiogenic, natural health products, herbal medicine, anticancer, clinical trials, integrative, molecular biology

## Abstract

An integrative approach for managing a patient with cancer should target the multiple biochemical and physiologic pathways that support tumour development and minimize normal-tissue toxicity. Angiogenesis is a key process in the promotion of cancer. Many natural health products that inhibit angiogenesis also manifest other anticancer activities. The present article focuses on products that have a high degree of anti-angiogenic activity, but it also describes some of the many other actions of these agents that can inhibit tumour progression and reduce the risk of metastasis. Natural health products target molecular pathways other than angiogenesis, including epidermal growth factor receptor, the *HER2*/*neu* gene, the cyclooxygenase-2 enzyme, the nuclear factor kappa-B transcription factor, the protein kinases, the Bcl-2 protein, and coagulation pathways. The herbs that are traditionally used for anticancer treatment and that are anti-angiogenic through multiple interdependent processes (including effects on gene expression, signal processing, and enzyme activities) include *Artemisia annua* (Chinese wormwood), *Viscum album* (European mistletoe), *Curcuma longa* (curcumin), *Scutellaria baicalensis* (Chinese skullcap), resveratrol and proanthocyanidin (grape seed extract), *Magnolia officinalis* (Chinese magnolia tree), *Camellia sinensis* (green tea), *Ginkgo biloba,* quercetin, *Poria cocos, Zingiber officinalis* (ginger), Panax ginseng, *Rabdosia rubescens* hora (Rabdosia), and Chinese destagnation herbs. Quality assurance of appropriate extracts is essential prior to embarking upon clinical trials. More data are required on dose–response, appropriate combinations, and potential toxicities. Given the multiple effects of these agents, their future use for cancer therapy probably lies in synergistic combinations. During active cancer therapy, they should generally be evaluated in combination with chemotherapy and radiation. In this role, they act as modifiers of biologic response or as adaptogens, potentially enhancing the efficacy of the conventional therapies.

## 1. INTRODUCTION

To progress, cancers require a source of nutrition and oxygen. Tumours that outgrow their oxygen supply cannot form masses greater than 1–2 mm without developing central necrosis. Neoplasms are genetically plastic and often adapt by switching on genes that increase their ability to invade and metastasize. A critical part of this process is the induction of local small blood vessels, termed “angiogenesis” 1,2.

Tumours do not grow progressively unless they induce a blood supply from the surrounding stroma. Cancers that lack angiogenesis remain dormant. Rapid logarithmic growth follows the acquisition of a blood supply. The tumour angiogenic switch seems to be activated when the balance shifts from angiogenic inhibitors to angiogenic stimulators.

The process of neovascularization is subtly controlled in normal tissues by a sequence of endogenous polypeptides that are secreted during growth, healing, and tissue renewal (Table I). Neoplasms are able to synthesize or induce some of these polypeptides, an activity that is partly achieved by the secretion of vascular endothelial growth factor (vegf) and angiopoietins (apns). Hypoxia stimulates these peptides; the result is a sprouting of endothelial cords. This sprouting creates profuse but immature networks of thin endothelial-lined channels, essential for tumour oxygenation. Although these networks permit progressive tumour growth, they are less efficient than the vascular supply to normal tissues. The apns recruit pericytes and initiate modelling of the vessel wall to more mature forms. Tumours often secrete a relative excess of vegf that results in disorganized and leaky vessels that cause local bleeding and edema.

Anti-angiogenic therapy has this theoretic attraction: it may be less susceptible to development of treatment resistance because it is directed to stroma rather than to genomically unstable tumour cells. Judah Folkman and his colleagues were the first to propose using inhibition of tumour vasculature formation as anticancer therapy 3. Their proposal led to the development and clinical trial of more than 20 drugs from groups that inhibit various steps in angiogenesis.

Recently, targeted therapies using monoclonal antibodies that antagonize the formation of new blood vessels have been developed. One example is bevacizumab (Avastin: Genentech, San Francisco, CA, U.S.A.). Bevacizumab is a genetically engineered humanized monoclonal immunoglobulin G antibody that blocks the vegf receptor in endothelial cells, thereby shutting off the tumour blood supply. When used in conjunction with chemotherapy, bevacizumab has been shown to extend life by a few months for some metastatic colorectal cancer patients 4. Preliminary evidence is available that adding bevacizumab to paclitaxel and carboplatin can improve survival by 2 months for non-squamous-cell lung cancer patients. Although bevacizumab increases survival for some patients, it increases the risk of adverse effects, including leucopenia, diarrhea, and hypertension. Use of bevacizumab is also associated with major risks of thrombosis (resulting in stroke and myocardial infarction), fatal hemorrhage (such as gastrointestinal bleeding or hemoptysis), and visceral perforation 5.

Anti-angiogenic therapies may also be combined with radiotherapy to improve local tumour control and reduce the risk of metastasis. During a course of radiotherapy, some tumours increase their angiogenic activity 6. Combined-modality therapy with anti-angiogenesis compounds induces a normal microvascular bed out of the disorganized tumour vessels. During the anti-angiogenic treatment, a critical time occurs when the vegf:apn ratio approximates normal. At that point, pericytes are recruited, the vascular basement membrane adopts a thinner morphology and tumour oxygenation temporarily increases. This is a favourable time to apply ionizing radiation, because such radiation is preferentially lethal to replicating and well-oxygenated cells. The combination of the anti-angiogenic agent and the radiation therapy is optimally effective if this window of opportunity is exploited 7–9.

So far, the evidence suggests that single anti-angiogenic agents have limited efficacy. Natural health products contain a range of complex organic chemicals that may have synergistic activity. They may inhibit angiogenesis by interacting with multiple pathways and by acting in other ways that can affect cell signalling, the apoptotic pathway, and the interaction of cancer cells with the immune system. Some anti-angiogenic agents also have anticoagulation activity that may also be associated with a reduction in metastasis. Heparin is a well-known example of a therapy with both anticoagulation and anti-angiogenic activities.

Rather than develop multiple monoclonal antibodies to target the multiple peptides and their receptors, an alternative approach might be to evaluate phytochemicals and certain animal-derived chemicals that influence multiple pathways. The science of pharmacognosy evaluates natural drugs derived from herbal remedies or phytomedicines. Minimal clinical research has been undertaken to evaluate the use of natural drugs as adjuvant therapy with conventional treatment using cytotoxic drugs and radiotherapy. Formal research is required on the timing of administration of natural health products with anti-cancer therapies. As noted in the earlier discussion of the administration of radiotherapy with anti-angiogenic treatment, timing may be critical. Anti-angiogenic natural health products may be most effective as maintenance therapy that impedes cancer recurrence following cytotoxic treatment. Human tumours can remain dormant for years because of a balance between cell proliferation and apoptosis.

## 2. WHAT IS ANGIOGENESIS?

Normal angiogenesis is the regulated formation of new blood vessels from existing ones. It is the basis of several physiologic processes, such as embryonic development, placenta formation, and wound healing. It is a good example of how a tumour can take control of normal processes and deregulate them to its own advantage.

In the normal and orderly formation of new blood vessels, endothelial cells receive a stimulatory signal from angiokinins and secrete specific enzymes such as matrix metalloproteinase (mmp) and heparinase that result in the dissolution of the extracellular matrix (ecm). The tight junctions between the endothelial cells are disrupted. The endothelial cells can then project through the newly created spaces and organize into fresh capillary tubes that grow toward the source of the blood supply 10,11.

The induction of new blood vessels provides tumours with a survival advantage. The survival and growth of cells depends on an adequate supply of oxygen and nutrients and on the removal of toxic products. Oxygen can diffuse radially from capillaries for only 150–200 μm. When distances exceed this maximum, cell death follows. Thus, the expansion of tumour masses beyond 1 mm in diameter depends on the development of a new blood supply—angiogenesis 12–14.

An increasing density of tumour vasculature raises the probability that the tumour will metastasize. Generally, increased microvascular density (“angiogenesis index”) is a significant indicator of poorer prognosis. Angiogenesis plays a central role in the progression of most solid tumours, including those of bladder, brain, breast, cervix, colon, lung, and prostate. Increased vascular density has also been found in the bone marrow of patients with acute myeloid leukemia and myeloma 15–24.

Cancer cells begin to promote angiogenesis quite early in the development of a tumour. The angiogenesis is characterized by oncogene-driven tumour expression of pro-angiogenic proteins (Table I).

The formation of new vasculature occurs in sequential steps. Endothelial cells must proliferate, migrate, and penetrate host stroma and the ecm. The endothelial cells must also undergo morphogenesis. The process of angiogenesis consists of activation and resolution phases. Activation requires initial degradation of the basement membrane, followed by endothelial cell migration, invasion of the surrounding ecm, endothelial cell proliferation, and capillary lumen formation. During resolution, the microvasculature matures and stabilizes by enclosure of the vessel by pericytes, inhibition of endothelial proliferation, reconstitution of basement membrane, and formation of gap junctions.

The vasculature of many solid tumours is not identical to that of normal tissue 25. The resolution phase is often incomplete in tumours, resulting in tumour microvessels that are highly irregular and tortuous and that are only partially lined with endothelium and basement membrane. Arteriovenous shunts and blind ends are common.

Failure of resolution may be a consequence of persistent overexpression of apn-2 in the tumour-associated vasculature. Differences are seen in cellular composition and permeability, in vessel stability, and in regulation of growth. The balance between factors that stimulate new blood vessel growth and those that inhibit it determines the vascular density. The inhibitory influence predominates in normal tissues; in tumours, many neoplastic cells switch from an angiogenesis-inhibiting to an angiogenesis-stimulating phenotype. That switch coincides with the loss of the wild-type allele of the *TP53* tumour suppressor gene and is associated with reduced production of thrombospondin-1 (tsp-1), a controller of angiogenesis in fibroblasts 26–31.

The production of vegf is considered essential for most cancer cell migration and for angiogenesis. A high vegf expression level is associated with worse outcome in a wide array of malignancies. Expression of vegf messenger rna is upregulated by many oncogenes (including H-*ras* and K-*ras*, *src*, *TP53*, and c-*jun*) and growth factors including epidermal growth factor, transforming growth factors alpha and beta, insulin-like growth factor–1, and platelet-derived growth factor 32–38. Table II lists some cancer-associated genes implicated in angiogenesis.

## 3. THE ANGIOGENIC–METASTATIC PATHWAY AS A TARGET FOR ANTICANCER THERAPIES

The process of cancer metastasis consists of a series of interrelated sequential steps. Each step is rate-limiting and may be a target for therapy. The outcome of the process depends on both the intrinsic properties of the tumour cells and the responses of the host. The balance of these interactions varies from one tumour and patient to another. These are the major steps 39–41 in the formation of a metastasis:

1. Transformation of normal cells into tumour cells, followed by growth. Initially depends on nutrients supplied by simple diffusion.
2. Extensive vascularization (angiogenesis). Vascularization must occur if the tumour mass is to exceed 1 mm in diameter. The production and secretion of pro-angiogenic factors by tumour cells and host cells plays a major role in establishing a capillary network from the surrounding host tissue.
3. Local invasion. Tumour cells use several mechanisms to invade the host stroma. Thin-walled venules, fragmented arterioles, and lymphatic channels offer little resistance to penetration and provide the most common pathways for entry of tumour cells into the circulation.
4. Detachment and embolization. Single cells or clumps break away. Most circulating tumour cells are rapidly destroyed. Those that survive must arrest in the capillary beds of distant organs by adhering either to capillary endothelial cells or to the exposed subendothelial basement membrane.
5. Extravasation into new host organ or tissue.
6. Proliferation within the new host organ or tissue. To continue growing beyond the 1-mm diameter, the micrometastasis must develop a vascular network and evade destruction by host defences. The cells can then continue to invade blood vessels, enter the circulation, and produce additional metastases.

The growth of many cancers is associated with an absence of the endogenous inhibitors of angiogenesis—for example, interferon beta (infβ). A potent inhibitor of angiogenesis, infβ works by blocking interleukin-8 (IL-8), basic fibroblast growth factor (bfgf), and collagenase type v, which are all potent angiogenic factors that aid tumour development and invasiveness.

Vascular endothelial growth factor stimulates the proliferation and migration of endothelial cells and induces plasminogen activity and the expression of metalloproteinases. In several animal models, overexpression of vegf in tumour cells enhances tumour growth and metastasis by stimulating vascularization 12,42–49.

Some cytotoxic chemotherapy agents are being used at lower-than-normal doses, with the intent of inhibiting angiogenesis and minimizing toxicity 50,51. This strategy may permit advanced cancer patients to maintain a better quality of life. This low-dose therapy is termed “metronomic dosing” 52–54.

The metronomic model of conventional cytotoxic chemotherapy suggests that advantages may also accrue to the administration of combinations of phytochemicals that interact with the multistep process of angiogenesis 55. In other words, targeting the vascular endothelium with continuous low-dose noncytotoxic therapies may maintain tumour control without excessive toxicity. The potential role of such therapies for increasing overall survival (but not necessarily disease-free survival) and for maintaining quality of life requires evaluation in future clinical trials.

### 3.1 Role of the Tumour Microenvironment in Mediating the Response to Anti-angiogenic Therapy

Each individual tumour may display a different angiogenic phenotype because the expression of angiogenic factors in tumours is controlled both by intrinsic factors in the tumour cell and by the influence of the host microenvironment 56. The microenvironment can effect gene expression in tumours growing at various sites. The tumour cells themselves can alter the endothelial cell phenotype. Various sites of metastasis may express varying combinations of angiogenic factors and endothelial cell phenotypes 57. Interactions among the polypeptide angiogenic factors produced by the tumour are complex, functioning in a dynamic, reciprocal fashion with other factors present in the tumour microenvironment. Therefore, cytokine-targeted anti-angiogenic therapies or monoclonal antibodies against angiogenic growth factors must consider not only the angiogenic factors that are being released by tumour cells, but also the contribution of the tumour microenvironment to tumour angiogenesis.

The efficacy of anti-angiogenic compounds varies from one tumour to another. The more specific the intervention is to one domain of the angiogenic pathway, the less likely a beneficial reduction in tumour growth is to occur, because alternative pathways can compensate. If the angiogenic activity of a tumour is initiated primarily by only one or two factors, then blocking the activity of one factor may be enough to inhibit tumour growth. For example, expression of vegf and epidermal growth factor receptor (egfr) correlate with the metastatic characteristics of human colon cancer, and so targeting vegf or egfr may be beneficial 58. However, if several factors mediate the angiogenic activity in a particular tumour, an alternative intervention strategy is required.

Natural health products contain a cocktail of biologic chemicals that act on multiple pathways that initiate and maintain tumour angiogenesis. In addition, we hypothesize that angiogenesis within the tumour microenvironment may be more sensitive to a cocktail of natural health products administered continuously at relatively low doses than to single-agent pharmaceutical compounds administered intermittently at higher dose levels. In general, as compared with normal tissues, tumours contain very immature blood vessels that may be relatively more susceptible to anti-angiogenic therapies, permitting a therapeutic gain 59,60.

### 3.2 Screening Herbs for Anti-angiogenic Activity

One of the first anti-angiogenic agents to be isolated was a phytochemical. In 1990, Ingber *et al.* reported on the anti-angiogenic properties of fumagillin, a secreted antibiotic of the fungus *Aspergillus fumigatus* 61. Refined fumagillin produces excess toxicity, and so analogues of fumagillin were subsequently synthesized.

Various assays are used to screen natural health products for anti-angiogenic activity 51,62,63. Assays used for screening are briefly discussed in the next few subsections.

#### *3.2.1* In Vitro *Assays*

The ability to maintain endothelial cells in culture has enabled the study of endothelial cell proliferation, migration, and function. For example, anti-angiogenic activity can be assessed by evaluating the potential of a substance to inhibit endothelial cell migration across a Boyden chamber. The bovine aortic endothelial cell assay is an established system.

*In vitro* assays are relatively inexpensive and give more rapid results. However, an ability to inhibit endothelial cell proliferation, migration, and tubule formation *in vitro* may not predict *in vivo* response. *In vitro* assays are a rapid method for initial screening of large numbers of agents. Definitive conclusions cannot be based on *in vitro* assays alone.

#### *3.2.2* In vivo *Assays*

*In vivo* biologic assays are more specific for detecting anti-angiogenic activity. The chick embryo chorioallantoic membrane model is an extra-embryonic membrane that is commonly used to study agents that influence angiogenesis. An angiogenic response in the form of increased vessel density around the implant occurs 72–96 hours after stimulation with an angiogenic compound. An angiostatic compound will induce the vessels around the implant to become less dense and even to disappear. Other systems include animal cornea implantation, disc angiogenesis, Matrigel (Becton–Dickinson, Mountain View, CA, U.S.A.) systems, and tumour xenograft models.

*In vivo* assays provide a more complete physiologic assessment of angiogenesis, but are more time-consuming and expensive.

### 3.3 Criteria for Anti-angiogenic Activity

The degree of anti-angiogenic activity is dose-dependent. Most chemotherapy drugs have anti-angiogenic activity when administered at high doses. Clinicians are especially interested in compounds that, when administered at low doses, specifically interact and antagonize the steps involved in angiogenesis. These agents may have relatively low toxicity at low doses and may exhibit a higher therapeutic gain.

Most conventional chemotherapy drugs have some degree of anti-angiogenic activity as a consequence of their cytotoxic activity. Ideal botanical derivatives would specifically antagonize new vessel formation in tumours without significant toxicity to normal tissues and without major adverse reactions. The ideal agent would also inhibit tumour cell proliferation through other physiologic pathways, such as intracellular signalling pathways.

Multiple levels of anti-angiogenic activity may be required to overcome the development of resistance by tumour-associated endothelial cells. Survival factors—such as increased secretion of vegf and bfgf by the tumour cells—activate intracellular pathways that prevent apoptosis in tumour-associated endothelial cells.

Maximal anti-angiogenic activity usually requires prolonged exposure to low concentrations of the active agent. This approach contrasts with the concept of administering maximum-tolerated doses of cytotoxic drugs to maximize tumour-cell kill. Some reports have confirmed the utility of combining low, frequent–dose chemotherapy with an agent that specifically targets the endothelial cell compartment 52,53. The evidence suggests that an anti-angiogenic schedule can be more effective than the use of high-dose cytotoxic drugs alone. We hypothesize that concomitant scheduling of anti-angiogenic botanicals with low, frequent–dose cytotoxic therapies may have biologic advantages that can increase therapeutic gain.

## 4. NATURAL HEALTH PRODUCTS THAT INHIBIT ANGIOGENESIS

Further research is necessary to screen herbs that may be useful anti-angiogenic therapies. Tables III and IV list natural health products with anti-angiogenic activity, and Table V lists herbs and their derivatives that inhibit vegf 55. A master herbalist can advise on potential herbal treatments derived from centuries of traditional observations and advanced traditional medical systems such as Traditional Chinese Medicine. It will be imperative to develop a new model of modern pharmacology based on traditional pharmacognosy.

Our developing knowledge of cancer biology suggests that administering cytotoxic drug therapy at very high doses is not always appropriate. A new approach is to administer lower doses of synergistic organic chemicals. These complexes already exist in myriad botanicals. New laboratory techniques permit more specific assays of activity, enabling maintenance of quality assurance and consistency between batches of botanical preparations. Such quality standards will permit credible clinical trials of anti-angiogenic natural health products to be initiated. At the same time, the importance of a holistic approach to managing the patient with cancer should not be minimized. Anti-angiogenic therapies form only a small part of a complex management program. Attention to the patient’s overall health and ability to mount an immune response are subtle factors that may become more important in tipping the balance towards cancer control.

### 4.1 Herbs and Phytochemicals

#### *4.1.1* Artemisia annua *(Chinese Wormwood)*

Artemisinin is the active constituent extracted from the plant *Artemisia annua.* Artemisinin has been used clinically as an anti-malaria drug 65. More recently, it was shown to be cytotoxic to cancer cells through induction of apoptosis 66.

Artesunate is a semi-synthetic derivative of artemisinin. Artesunate was tested *in vitro* in the human umbilical vein endothelial cell (huvec) model of angiogenesis and was shown to significantly inhibit angiogenesis in a dose-dependent manner 67. The inhibition of proliferation of huvecs was greater than that seen with cancer cells, fibroblast cells, and human endometrial cells. Those findings indicate that the anti-angiogenic activity of artesunate is greater than its cytotoxicity.

The anti-angiogenic effect of artemisinin *in vivo* was evaluated using transplanted human ovarian cancer (HO-891) cells in nude mice. Immunohistochemical staining for microvessel CD31 antigen, vegf, and the vegf receptor (*KDR*, formerly called *FLK1*) was performed. In treated mice, tumour growth was decreased and microvessel density was reduced without any toxicity to the host animals. Artemisinin also lowered vegf expression by tumour cells and *KDR* expression by endothelial cells. Artemisinin also has anticancer activity through other pathways. It inhibits the activation of nuclear factor kappa-B (nf-κb), an important activator protein in cancer development and progression 68.

#### *4.1.2* Viscum album *(European Mistletoe)*

*Viscum album* is also known as Iscador (Weleda, Palisades, NY, U.S.A.). It is often used as an anticancer agent in anthroposophic and homeopathic medicine. Laboratory studies show that it is anti-angiogenic by downregulation of vegf; it also induces apoptosis of cancer cells 69,70. In a mouse model, lung metastases were reduced, and survival was increased 71. A clinical trial in human subjects showed an increase in survival in a variety of cancers, but the study was poorly controlled and no definitive conclusions could be drawn 72. Well-controlled clinical trials of *V. album* derivatives in combination with other anticancer therapies are warranted.

#### *4.1.3* Curcuma longa *(Curcumin)*

Curcumin is the most active curcuminoid in turmeric. It interacts with cancer cells at a number of levels and can enhance the tumoricidal efficacy of cytotoxic chemotherapy and radiotherapy 73–75. Its anti-invasive effects are partly mediated by downregulation of matrix metalloproteinase-2 (*MMP2*) and upregulation of tissue inhibitor of metalloproteinase-1 (*TIMP1*) 76. These enzymes are involved in the regulation of tumour cell invasion.

Curcumin inhibits the transcription of two major angiogenesis factors, vegf and bfgf 77. It interacts with vegf- and nitric oxide–mediated angiogenesis in tumours 78,79. Elevated levels of nitric oxide correlate with tumour growth. Curcumin reduces nitric oxide generation in endothelial cells. The membrane-bound enzyme CD13 (aminopeptidase N) is found in blood vessels undergoing active angiogenesis. Curcumin binds to CD13 and blocks its activity, thereby inhibiting angiogenesis and invasion by tumour cells 80,81. Derivatives of curcumin may be developed to target CD13, providing a novel approach to reduce neoplastic angiogenesis 82,83.

Curcumin also downregulates the expression of the *VEGF* and *MMP9* genes that are associated with angiogenesis. Demethoxycurcumin is a structural analogue of curcumin isolated from *Curcuma aromatica.* It specifically inhibits the expression of *MMP9* 84. Curcumin can interfere with the activity of both *MMP2* and *MMP9,* the basis of the angiogenic switch, thereby reducing degradation of the ecm 85. It also interferes with the release of angiogenic factors that are stored in the ecm. It inhibits growth factor receptors such as egfr and vegf receptor and the intracellular signalling tyrosine kinases. This cell signalling system can promote further angiogenesis through gene activation that increases levels of cyclooxygenase-2 (cox-2), vegf, il-8, and the mmps^86^–^88^.

A phase i study of curcumin found no treatment-related toxicity at doses up to 8000 mg daily. Beyond 8000 mg daily, the bulky volume of the drug was unacceptable to the patients. Serum concentration of curcumin usually peaked at 1–2 hours after oral intake of curcumin and gradually declined within 12 hours 89. The study suggested that curcumin may prevent cancer progression. Derivatives of curcumin, such as copper chelates of curcuminoids, may have increased antitumour activity 83.

#### *4.1.4* Scutellaria baicalensis *(Chinese Skullcap)*

Baicalin and baicalein are the main derivatives of the Chinese skullcap herb. They are potent anti-angiogenic compounds that reduce vegf, bfgf, 12-lipoxygenase activity, and mmp 90,91. *Scutellaria baicalensis* is one of the herbs found in pc-spes, a complex of Chinese herbs that is clinically active against advanced prostate cancer 92–94.

#### 4.1.5 Resveratrol and Proanthocyanidin (Grape Seed Extract)

Resveratrol is a phytoalexin found in grapes and wine. It has anti-angiogenic activity demonstrated by its ability to inhibit division in huvecs and to decrease the lytic activity of mmp-2 95. Resveratrol inhibits vegf-induced angiogenesis by disruption of reactive oxygen species–dependent *src* kinase activation and subsequent ve-cadherin tyrosine phosphorylation 96,97. Resveratrol inhibits the growth of gliomas in rats by suppressing angiogenesis 98.

Edible berries contain high concentrations of proanthocyanidin. The latter inhibits vegf expression induced by tumour necrosis factor alpha (tnfα). Feeding proanthocyanidins to mice with tumour xenografts reduced vegf secretion, which resulted in reduced intratumoral microvasculature 99–101. On the other hand, one study showed that grape seed extract may upregulate oxidant-induced vegf expression, suggesting that proanthocyanidin can induce angiogenesis as part of normal tissue healing 102.

#### *4.1.6* Magnolia officinalis *(Chinese Magnolia Tree)*

The seed cones of the Chinese magnolia tree contain substances that inhibit the growth of new blood vessels. Honokiol is the active ingredient. It may partly reduce angiogenesis through the regulation of platelet-derived endothelial cell growth factor and transforming growth factor beta (tgfβ) expression. It also inhibits nitric oxide synthesis and tnfα expression 103,104. In animal experiments, honokiol suppressed proliferation in blood vessel endothelial cells more than in other types of cells and thereby reduced tumour growth 105,106.

#### *4.1.7* Silybum marianum *(Milk Thistle)*

Silibinin and silymarin are polyphenolic flavonoids isolated from the fruits or seeds of *Silybum marianum.* In the laboratory, silymarin demonstrates strong activity against a variety of tumours by downregulation of vegf and egfr 107,108. Silymarin suppresses vegf when used as a single agent against human ovarian cancer 109.

#### *4.1.8* Camellia sinensis *(Green Tea)*

Tea contains polyphenols and catechins, mainly epigallocatechin-3 gallate (egcg) 110. These constituents inhibited proliferation of MDA-MB231 breast cancer cells and huvecs 111 and, in rodent studies, also suppressed breast cancer xenograft growth and reduced the density of tumour vessels 112. This activity was associated with a decrease in vegf, regulated at the level of transcription. In addition, egcg suppresses protein kinase C (pkc), another vegf transcription modulator.

Inhibition of vegf transcription is one of the molecular mechanisms involved in the anti-angiogenic effects of green tea that may contribute to its potential use for cancer treatment 113,114. Epigallocatechin-3 gallate may be administered as a powdered extract of green tea. An appropriate dose has been extrapolated from anti-angiogenic activity in rodent experiments^115^ as well as from a phase i study in humans 116. A dose of 1.0 g/m^2^ three times daily [equivalent to 7–8 Japanese cups (120 mL)] has been recommended. In practice, lower total daily doses of 2–4 g standardized green tea extract (95% polyphenols and 60% catechins) are usually prescribed. Each gram of this extract provides 400–500 mg of egcg. The dose-limiting adverse effects are the gastrointestinal and neurologic effects of caffeine. However, the caffeine may potentiate the anti-angiogenic effect of egcg 116.

#### *4.1.9* Ginkgo biloba

*Ginkgo biloba* extract has anticancer effects that are related to its gene-regulatory and anti-angiogenic properties. The *Ginkgo biloba* extract used in most research is EGb 761, which contains about 25% flavonoids (ginkgo-flavone glycosides) and about 5% terpenoids (ginkgolides and bilobalides). The most potent flavonoid is ginkgolide B. The extract inhibits angiogenesis by downregulating vegf 117,118.

#### 4.1.10 Quercetin

Quercetin is a flavone found in apples, onions, raspberries, red grapes, citrus fruit, cherries, broccoli, and leafy greens. It inhibits angiogenesis through multiple mechanisms, including interaction with the cox-2 and lipoxygenase-5 enzymes, egfr, the *HER2* intracellular signalling pathway, and the nf-κb nuclear transcription protein 119–123. Quercetin may enhance the anticancer effects of tamoxifen through anti-angiogenesis 124.

#### *4.1.11* Poria cocos

*Poria cocos* is a mushroom extract that, by tradition, has anticancer activity. It inhibits platelet aggregation and appears to be anti-angiogenic by down-regulating nf κb 125–129

#### 4.1.12 Panax Ginseng

The lipophilic constituents of ginseng are called saponins (or ginsenosides). These extracts possess anticancer activities in tumours that include anti-angiogenesis and induction of tumour cell apoptosis 130.

#### *4.1.13* Rabdosia rubescens *Hara (Rabdosia)*

Rabdosia is used in certain traditions to treat cancer. It is one constituent of the pc-spes formula that is active against prostate cancer. It contains ponicidin and oridonin, two diterpenoids that possess significant anti-angiogenic activity 131.

#### 4.1.14 Extracts of Chinese Medicinal Herbs

Herbs that are used by tradition in China as anticancer agents have been screened for their anti-angiogenic activity 62. Table VI lists the most active herbs (those that exhibit more than 20% inhibition at 0.2 g herb/mL) by chorioallantoic membrane and bovine aortic endothelial cell assays.

### 4.2 Copper Antagonists

Some cancers are associated with high serum levels of copper. The role of copper in cancer promotion through pro-inflammatory cascades and angiogenesis induction is quite well established 132. Copper is essential for the function of many angiogenic growth factors. The angiogenic activity of bfgf, vegf, tnfα, and il-1 are copper-dependent.

Copper chelation with tetrathiomolybdate is a promising therapy for tumour control 133,134. The hypothesized mechanism of action for this substance is inhibition of angiogenic cytokines. Unlike certain current approaches to anti-angiogenic therapy that target single agents, tetrathiomolybdate inhibits multiple angiogenic cytokines. Part of the effect appears to stem from inhibition of nf-κb, which in turn controls transcription of many angiogenic factors and other cytokines.

Some angiogenic cytokines appear to have separate mechanisms of copper dependence. The inhibition of multiple angiogenic cytokines gives tetrathiomolybdate the potential to be a more global inhibitor of angiogenesis. Several aromatic herbs—such as *Caryophylli flos, Cinnamomi* cortex, *Foeniculi fructus,* and *Zedoariae rhizoma—*have inhibitory effects on lipid peroxidation or protein oxidative modification by copper 135. They may have a role to play in anti-angiogenesis, but further research is necessary for confirmation.

### 4.3 Animal Products

#### 4.3.1 Shark and Bovine Cartilage

The resistance of cartilage to tumour formation has been correlated with its capacity to inhibit the formation of new blood vessels. A number of *in vitro* and *in vivo* studies have suggested the existence of anti-angiogenic compounds in shark and bovine cartilage 136. The clinical effectiveness of whole cartilage for the treatment of cancer was not confirmed in a recent phase iii randomized controlled trial 137. The main problem is lack of data correlating bioavailability with pharmacologic effects in the oral use of shark cartilage. Unsatisfactory outcomes in clinical trials may be secondary to inadequate bioavailability of the active constituents 138. Bioactive derivatives of shark cartilage are being extracted. The AE-941 derivative (Neovastat: Æterna Zentaris, Quebec, QC, Canada) is a standardized water-soluble extract that represents less than 5% of the crude cartilage. This multifunctional anti-angiogenic product contains several biologically active molecules 139. The mode of extraction developed by Æterna differs from that of many other preparations and may explain the preservation of the anti-angiogenic properties.

Neovastat is kept frozen until use to maximally preserve its biologic properties. Its anti-angiogenic activity may be attributable to the presence of a metalloproteinase inhibitor with a preferential inhibition of mmp-2 and to inhibition of serine elastase, of vegf binding to endothelial cells, and of tyrosine phosphorylation of the vegf receptor. Neovastat reduces the vegf-dependent increase in vascular permeability.

Paradoxically, shark cartilage extracts (including AE-941) also have fibrinolytic activity 140,141. However, fibrinolysis and anticoagulation may also reduce tumour cell metastasis 142,143. Shark cartilage extracts are pleiotropic, having multiple phenotypic activities.

No published phase iii randomized controlled trials have yet proven the utility of Neovastat for cancer treatment. Part of the funding for clinical studies of AE-941 comes from Technology Partnerships Canada (tpc), a research support program run by Canada’s federal government. The agreement is that Æterna will reimburse tpc upon commercialisation of AE-941–derived products. In January 2004, Æterna announced that development of AE-941 would be focused on non-small-cell lung cancer only 144.

#### *4.3.2* Squalus acanthias *(Dogfish Shark)*

Squalamine is a cationic steroid isolated from the liver of the dogfish shark, *Squalus acanthias* 145. Squalamine significantly blocks vegf-induced activation of mitogen-activated protein kinase and cell proliferation in human vascular endothelial cells. Squalamine is anti-angiogenic for ovarian cancer xenografts, and it appears to enhance the cytotoxic effects of cisplatin chemotherapy, independent of *HER2* status. Overexpression of *HER2* is normally associated with resistance to cisplatin and promotion of tumour angiogenesis 146. In a phase ii trial of patients with advanced small-cell lung cancer, squalamine was administered at a dose of 300 mg/m^2^ by continuous infusion for 5 days, with paclitaxel and carboplatin given on day 1. Patient survival data and a satisfactory safety profile indicated that the combination should be explored further 147.

## 5. CONCLUSION

*In vitro* and *in vivo* studies are uncovering anti-angiogenic activity in many natural health products. Further preclinical research is required to define whether single compounds or complex mixtures will be optimal for clinical trials. A potential advantage of phytochemicals and other compounds derived from natural health products is that they may act through multiple cell-signalling pathways and reduce the development of resistance by cancer cells. Part 2 of this review will further discuss the latter issues.

## Figures and Tables

**Table I:** Endogenous angiogenic polypeptides

Activator protein 1 (ap-1)
Angiogenin (ag) and angiotropin (at)
Angiopoietin (apn)
Basic fibroblast growth factor (bfgf)
Cyclooxygenase (cox) and lipoxygenase (lox)
Granulocyte-colony stimulating factor (g-csf)
Hepatocyte growth factor (hgf)
Insulin-like growth factors 1 and 2 (igf-1 and -2)
Interleukin-8 (IL-8)
Nuclear factor kappa B (nf-κB)
Placental growth factor (pgf)
Platelet-derived endothelial cell growth factor (pd-ecgf)
Pleiotrophin (ptn)
Proliferin Thrombospondin-1 (tsp-1)
Transforming growth factor alpha (tgfα)
Transforming growth factor beta (tgfβ)
Tumour necrosis factor alpha (tnfα)
Vascular endothelial growth factor (vegf)
Vascular permeability factor (vpf)

**Table II:** Cancer-associated genes implicated in angiogenesis

*Oncogene*	*Growth factors or cytokine levels*^a^
H-/K-*ras*	vegf ↑, tsp-1 ↓, bfgf ↑
*src*	vegf ↑
*erb2*/*HER2*	vegf ↑, tsp-1 ↓
*EGFR*	vegf ↑, il-8 ↑, bfgf ↑
*HPV16*	vegf ↑
*BCR*–*ABL1*	vegf ↑
n-*myc*/c-*myc*	vegf ↑, tsp-1 ↓
*TP53*	vegf ↑, tsp-1 ↓
c-*jun*	vegf ↑, tsp-1 ↓

a See Table I for full names of these polypeptides.

↑ = increased level; ↓ = decreased level.

**Table III:** Natural health products with potential direct and indirect anti-angiogenic activity a

Herbs and associated phytochemicals
*Aloe barbadensis* (aloe vera leaf and pulp extracts)
*Angelica sinensis* (aqueous extracts)
*Artemisia annua* (artemisinin)
*Camellia sinensis* (epigallocatechin)
*Chrysobalanus icaco* (methanol extract)
*Curcuma longa* (curcumin)
*Dysoxylum binectariferum* (flavopiridol)
Flos magnoliae (magnosalin)
*Ganoderma lucidum* (triterpenoids)
*Ginkgo biloba* (ginkgolide B)
*Glycyrrhiza glabra* (isoliquiritigenin,; glabridin)
*Hibiscus sabdariffa* L. (protocatechuic acid)
*Livistona chinensis* (aqueous extract from seed)
*Matricaria chamomilla* (flavonoids: apigenin, fisetin)
*Ocimum sanctum* (carnosol, ursolic acid)
Omega-3 fatty acids (eicosapentaenoic acid, docosahexaenoic acid)
*Magnolia obovata* (honokiol)
Panax ginseng (saponins: 20(R)- and 20(S)-ginsenoside-Rg3)
*Polypodium leucotomos* (difur)
*Poria cocos* (1–3-α-d-glucan)
*Polygonum cuspidatum* (resveratrol)
Proanthocyanidin
Quercetin
*Rabdosia rubescens* Hora (ponicidin and oridonin)
*Rosmarinus officinalis* (carnosol and ursolic acid)
*Scutellaria baicalensis* (baicalin and baicalein)
*Silybum marianum* (silymarin)
Soy isoflavones (genistein, daidzein)
*Tanacetum parthenium* L. (parthenolide)
*Tabebuia avellanedae* (β-lapachone)
*Taxus brevifolia* (taxoids)
*Viscum album* (lectins)
*Zingiber officinale* (6-gingerol)
Other Chinese herbs (see Table VI)
Cyclo-oxygenase-2 antagonists (see Table IV)
Minerals
Selenium
Animal-derived
Bovine cartilage
Shark cartilage (water soluble extract AE-941)
Squalus acanthias (dogfish liver: squalamine)
Vitamin D (1α ,25-D_3_)

a Data derived from *in vitro* and *in vivo* studies cited in text.

**Table IV:** Natural health products that inhibit cyclo-oxygenase-2 activity^64^

Ginger
Aloe vera
Epigallocatechin-3 gallate/green tea
Resveratrol
Liquorice
Garlic
Chinese skullcap
Bilberry
Grape seed extract proanthocyanidins
Panax ginseng
Milk thistle
Fish oils: omega-3 fatty acids (eicosapentaenoic acid, docosahexaenoic acid)
Green-lipped mussel
Antioxidants (vitamins A, C, E; Se, Zn; carotenoids, flavonoids, coenzyme Q10, *N*-acetylcysteine, lipoic acid)
Boswellia
Bromelain
Curcumin
Quercetin

**Table V:** Herbs and their derivatives that specifically inhibit vascular endothelial growth factor and have direct activity against angiogenesis a

*Artemisia annua* (Chinese wormwood)	Contains 95% artemisinin, and other related terpenes and flavonoids
*Viscum album* (European mistletoe)	Contains mistletoe lectin iii (ML3A)
*Curcuma longa* (turmeric)	Contains 95% curcumin
*Camellia sinensis* (green tea)	Contains 95 % phenols; 50% epigallocatechin
*Vitis vinifera* (grape seed extract)	Contains 95% proanthocyanidins
*Angelica sinensis* (dong quai)	Contains 4-hydroxyderricin
*Taxus brevifolia* (Pacific yew)	Contains Taxol
*Scutellaria baicalensis* (Chinese skullcap)	Contains 95% baicalin and flavonoids
*Polygonum cuspidatum* (Japanese knotweed)	Contains 20% resveratrol
*Silybum marianum* (milk thistle)	Contains 80% silymarin (silybin)
Magnolia seed cones	Contains 90% honokiol
Other Chinese herbs (see Table VI)	

a Data derived from *in vitro* and *in vivo* studies cited in text.

**Table VI:** Anti-angiogenesis activity of Chinese medicinal herbal extracts (exhibiting more than 20% inhibition at 0.2g herb/mL)^62^

		*% Inhibition*	
*Name*	*Part used*	*cam*	*baec*
*Berberis paraspecta*	Root	25	38
*Catharanthus roseus*	Leaf	27	30
*Coptis chinensis*	Rhizome	25	37
*Scrophularia ningpoensis*	Root	20	34
*Scutellaria baicalensis*	Root	27	41
*Polygonum cuspidatum*	Whole plant	—	28
*Taxus chinensis*	Bark	—	26

cam = chick embryo chorioallantoic membrane assay; baec = bovine aortic endothelial cell culture assay.
